# ASEAN’s response to mpox: Advancing regional and inter-regional cooperation to manage public health emergencies

**DOI:** 10.1371/journal.pgph.0003985

**Published:** 2025-01-02

**Authors:** Afifah Rahman-Shepherd, Ahmed Razavi, Ifedayo Adetifa, Ngozi Erondu, Soawapak Hinjoy, Zheng Jie Marc Ho, Irvin Miranda, Ebere Okereke, Oyeronke Oyebanji, Justin Wong, Ferdinal Fernando

**Affiliations:** 1 Saw Swee Hock School of Public Health, National University of Singapore, Singapore, Singapore; 2 UK Health Security Agency, Jakarta, Indonesia; 3 Foundation for Innovative New Diagnostics, Geneva, Switzerland; 4 O’Neill Institute for National and Global Health Law, Georgetown University, Washington, D.C., United States of America; 5 Department of Disease Control, Ministry of Public Health, Bangkok, Thailand; 6 Asia Centre for Health Security, Singapore, Singapore; 7 Department of Health, Manila, Philippines; 8 Reaching the Last Mile Foundation, Nairobi, Kenya; 9 London School of Hygiene and Tropical Medicine, London, United Kingdom; 10 Ministry of Health, Bandar Seri Begawan, Brunei Darussalam; 11 Health Division, ASEAN Secretariat, Jakarta, Indonesia; PLOS: Public Library of Science, UNITED STATES OF AMERICA

## Introduction

Since the COVID-19 pandemic, the value of regional cooperation and the role regional organizations play in managing public health emergencies has received greater attention [1–4]. In this opinion, we present the Association of Southeast Asian Nations’ (ASEAN) response to mpox, highlighting the priorities for regional organizations across the health emergency management cycle. Bringing together perspectives from Southeast Asia and Africa, we further explore how organizations can advance cooperation at both regional and inter-regional levels to improve the management of mpox and future public health emergencies.

### ASEAN’s response to mpox

As a regional organization, ASEAN promotes and facilitates cooperation among its Member States to collectively manage threats to regional security. We highlight three critical functions of ASEAN’s role in responding to the public health emergency of international concern (PHEIC) for mpox in 2024 (Table 1).

**Table 1:** ASEAN mechanisms mobilized in response to mpox.

Mechanism	Response activities
ASEAN Emergency Operations Center Network	The Network is led by the Ministry of Health, Malaysia, and has been monitoring the mpox situation in the region. It disseminated critical information and convened all Member States through a webinar to facilitate the sharing of experiences, lessons learned, and best practices in managing health emergencies, including mpox; and to exchange information on effective strategies for outbreak response, surveillance, and risk communication between ASEAN and key public health organizations in Africa.
ASEAN Biodiaspora Virtual Center	The Center is led by the Ministry of Health, Indonesia, and conducts horizon scanning of emerging threats and aggregate risk assessment for the region. The Centre has provided Member States with regular intelligence reports on the current status of mpox cases within and beyond ASEAN.
COVID-19 ASEAN Response Fund	The Fund was established in 2020 in response to the COVID-19 pandemic, financed by existing cooperation funds and voluntary contributions from Member States. The Ministry of Health, Brunei Darussalam, submitted a proposal to mobilize the Fund to pool procurement of antiviral countermeasures for the region and is leading this initiative on behalf of the region.

First, its ability to rapidly convene and alert all ASEAN Member States following the first imported case of mpox Clade 1b reported in Thailand on 22 August 2024. The ASEAN Emergency Operations Centre (EOC) Network hosted a webinar attended by all Member States on 3 September, leveraging its unique convening power to promote regional solidarity and establish multilateral dialogue on response efforts.

Second, its efforts to pool procurement of countermeasures. ASEAN quickly mobilized the funds required for a one-off pooled procurement of antiviral vaccines and therapeutics. To date, eight Member States have participated in this pooled procurement strategy.

Third, its value as a platform for information and knowledge exchange. The EOC Network disseminated a press release about the first case of mpox from the Ministry of Public Health, Thailand, via WhatsApp within 24 hours of the national investigation team’s confirmation. Critically, the Director General of Africa CDC and the National Mpox Incident Manager of Nigeria CDC were invited to present, fostering shared learning from the African continent to ASEAN. The ASEAN Biodiaspora Virtual Centre has also provided regular risk assessments of mpox, tracking the spread of the virus.

### Key priorities for ASEAN in managing public health emergencies

**Fig 1:**
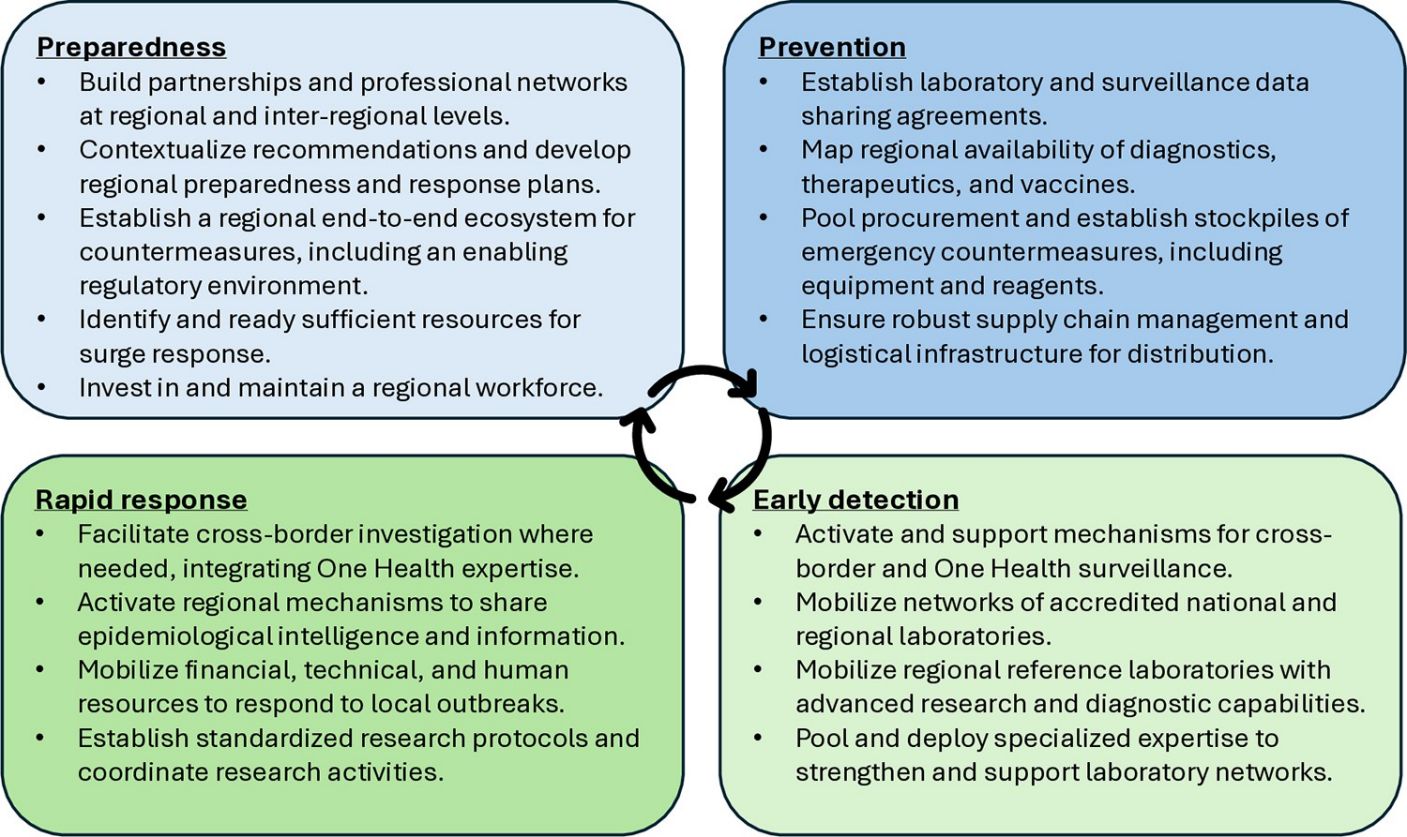
Recommended priorities for regional organizations in responding to public health emergencies. Managing public health emergencies is complex and non-linear, and we recognize that phases often overlap. This figure is for illustrative purposes only.

In anticipation of imported cases and localized outbreaks in their respective regions, we propose priorities for regional organizations across the emergency management cycle (Fig 1).

The ASEAN response highlights some of these priorities, but it has also underscored the need to fully operationalize the ASEAN Centre for Public Health Emergencies and Emerging Diseases to strengthen capabilities across the emergency management cycle [5, 6]. The Centre was announced in 2020 to strengthen the three pillars of Prevention and Preparedness, Detection and Risk Assessment, and Response and Risk Communications. Centres are hosted by Vietnam, Indonesia, and Thailand respectively, but as of December 2024, the Establishment Agreement has yet to be signed.

Whilst pooled procurement mechanisms reduce costs by aggregating demand, different regulatory standards and processes pose a challenge to rapidly and collectively procure countermeasures [7]. Much like the recently established African Medicines Agency, ASEAN could play a greater role in harmonizing regulatory systems. In terms of early detection, a key focus for ASEAN has been strengthening regional surveillance capabilities. In response to mpox, the ASEAN Health Cluster 2 facilitated direct collaboration with the Ministry of Public Health, Thailand, and key development partners to develop rapid serological and molecular tests capable of distinguishing between mpox Clades. These efforts can boost regional laboratory networks for quicker and more accurate diagnosis, as well as enhance the capacity of healthcare workers through targeted training and resource allocation.

Though there were efforts to coordinate a case series on all mpox cases in the region during the first PHEIC in 2022, it was a challenge for some Member States to share such detailed epidemiological intelligence. Issues around data security and privacy related to disease notification, as well as approval delays in formal notification pathways, mean that the information shared is not always actionable when it eventually arrives. Establishing agreements and protocols to share data during health emergencies would facilitate information and knowledge exchange, enhance regional surveillance activities and enable much needed research [8].

### Opportunities for inter-regional cooperation in public health emergencies

Building partnerships at an inter-regional level may have mutual benefits across the emergency management cycle [9, 10]. As demonstrated by the efforts of the ASEAN EOC Network, cooperation could focus on dialogue and knowledge exchange on best practices in key areas such as: diagnostics, epidemiological investigation, contact tracing, risk communications, and research. This presents an opportunity for regions further along in their responses to help enhance responses in other regions, and control issues such as mis- and disinformation through effective community-based risk communications. Ongoing partnerships between regional public health organizations, such as Africa CDC and ASEAN, could play an enabling role for such inter-regional exchange, and should not be limited to emergencies.

Beyond such exchanges, inter-regional cooperation could facilitate sharing of critical resources such as: testing kits, genetic material, medical countermeasures, and technical expertise [11]. Deploying technical experts between and within regions to manage outbreaks at their source could help with controlling further spread of the PHEIC to new regions and countries. Regional organizations can leverage expertise that is more developed in one region compared with another, for example, African expertise on managing mpox could be helpful to other regions.

Additionally, inter-regional cooperation could be used to facilitate progress on issues that appear to be bottlenecked at a global level. For instance, ASEAN Member States have a larger capacity for drug, diagnostics, therapeutics and vaccine manufacturing than African states. Technology transfer could help capacitate regions to be more self-sufficient in responding to PHEICs. This also applies to the licensing of new products. Up until September 13, 2024, Gavi and UNICEF were unable to bulk purchase mpox vaccines as no vaccinations had been authorised by the WHO Prequalification of Medical Products system [12]. Regional or inter-regional capacity to license such products may provide a way to bypass such bottlenecks, increasing speed and access to medical countermeasures in affected regions.

Inter-regional cooperation, while valuable, requires a careful balance with resource allocation. Dialogue and knowledge exchange is less resource intensive and can be a productive way to develop collaboration. However, mobilising financial and technical support may necessitate specific governance arrangements, the feasibility of which could depend on broader issues such as the geopolitical environment. Such arrangements must serve regional priorities and avoid overshadowing arrangements between national public health institutes. They should also complement, not duplicate, WHO’s coordination efforts globally, focusing on areas where inter-regional cooperation can add value.

## Conclusion

The global architecture for managing health emergencies is evolving, and regional organizations are playing an increasingly important role in facilitating greater coordination and cooperation amongst their Member States. As one of the first regions to confirm an imported case of mpox Clade 1b, ASEAN rapidly convened all Member States, enabled the exchange and sharing of critical information in a timely manner, and activated mechanisms to continuously monitor the situation and pool procurement of countermeasures. It has also demonstrated the value of inter-regional cooperation between ASEAN and key public health organizations in Africa with the knowledge and expertise in managing mpox. Though there are challenges to strengthening regional cooperation and further developing inter-regional partnerships, there are also important opportunities for regional organizations to add value and resilience to the architecture for health emergencies.
